# Open-Label Observational Study of a Topical Formulation of Calcium Spirulan Contained in a Defined Extract of the Microalga *Spirulina platensis* in the Treatment of Children with Molluscum Contagiosum

**DOI:** 10.1155/2023/8871299

**Published:** 2023-08-02

**Authors:** Karoline Jungclaus, Rosa Mascarenhas, Oscar Tellechea, Jeremias L. K. Reich, Kristian Reich

**Affiliations:** ^1^Center for Translational Research in Inflammatory Skin Diseases, Institute for Health Services Research in Dermatology and Nursing, University Medical Center Hamburg-Eppendorf, Hamburg, Germany; ^2^Hospital Distrital da Figueira da Foz, Dermatologia, Portugal; ^3^Oxford University Clinical Academic Graduate School, Oxford University, Oxford, UK

## Abstract

**Background:**

Molluscum contagiosum (MC) is a common viral skin infection primarily affecting children which is difficult to treat using available therapeutic approaches. The sulfated polysaccharide named calcium spirulan (Ca-SP) has demonstrated antiviral effects against herpes simplex virus in keratinocytes *in vitro*, and a cream containing 1.5% Ca-SP and 1% of a defined microalgae extract (Spiralin^®^) effectively prevented herpes labialis in a trial with susceptible individuals. This observational study aimed to show antiviral effects of a similar formulation (Spirularin^®^ VS) against MC in children.

**Methods:**

Children with active MC lesions were treated with Spirularin^®^ VS cream twice daily on affected skin over several months and asked to return for follow-up visits after 1 to 3 months. Clinical status of MC infection was documented at baseline and follow-up visits.

**Results:**

Of the 31 children enrolled in the study, 26 completed treatment and returned for control visits. Spirularin^®^ VS cream was applied twice daily over a period of 1 to 9 months (mean treatment duration 3.9 months). 19/26 (73.1%) children achieved complete clearance of MC lesions with no clinical evidence of bacterial skin infection during treatment. No irritative skin reactions or unpleasant symptoms were observed or reported.

**Conclusion:**

This open-label observational study suggests that a cream formulation containing 1.5% Ca-SP and 1% Spiralin® may be an effective and safe treatment option for children with active MC lesions. The high rate of complete clearance of MC lesions and lack of adverse reactions warrant further investigation in larger, controlled trials.

## 1. Background

Molluscum contagiosum (MC) is an epidermal infection occurring in humans worldwide caused by the double-stranded DNA molluscum contagiosum virus (MCV) of the Poxviridae family. The infection mostly occurs in children with a peak between 2 and 5 years. The average prevalence in persons aged 1 to 16 years is approximately 5% to 8% with a trend for a higher prevalence in regions with warmer climate [1]. Some studies indicate a much higher risk of MC in individuals with atopic dermatitis [2, 3], especially among carriers of a filaggrin mutation [4]. Sexually active adults and immunocompromised individuals can also be affected [5, 6]. MC is transmitted by direct skin contact, sexual or nonsexual, from one infected person to another or within an affected individual from one infected body part to another (autoinoculation). Transmission is also believed to occur indirectly via contaminated towels or water, e.g., in bathrooms, swimming pools, or saunas [7]. Highly contagious lesions typically present as smooth, glassy, pink, or skin-colored, dome-shaped papules of 2 to 5 mm in size[7, 8].

In children, the exposed skin areas are typically affected including the extremities and the face and also the trunk and intertriginous regions and the genitals (by autoinoculation), but not the palms and soles [9]. In adults, MC lesions are commonly found on the lower abdomen as well as on the genital and perianal areas.

Because MC lesions are often self-limiting within several months [8], the need for active treatment in cases with limited disease has been discussed controversially [7, 8, 10, 11]. Likely considering the risk of progression to more extensive disease and possible complications such as bacterial superinfections and the development of eczematous lesions, however, most dermatologists, pediatricians, and patients (and their caregivers) prefer active treatment [8, 10, 11]. Various mechanical, chemical, immunomodulatory, and antiviral treatment modalities have been used, but until today, no single intervention has convincingly been shown to be effective and well tolerated in the treatment of MC [12].

A sulfated polysaccharide, termed calcium spirulan (Ca-SP), contained in a defined extract from the microalga *Spirulina platensis* (Spiralin^®^) has been shown to exert antiviral effects against herpes simplex virus infections of human keratinocytes by interfering with the viral attachment to proteoglycans on the host cell surface, and a Ca-SP and Spiralin^®^ containing topical formulation effectively prevented herpes labialis in a clinical trial with susceptible individuals [13]. We here report the results of an open-label observational study with a similar formulation in children with active MC.

## 2. Methods

An open-label observational study of a topical formulation containing 1.5% Ca-SP and 1% Spiralin^®^ was conducted in Portuguese children below the age of 16 years. The specific formulation of this cosmeceutical has been approved as Spirularin^®^ VS cream in Portugal since December 2013 and is similar to the formulation previously described [13]. Caregivers and their children attending a single Portuguese children's hospital with active MC lesions were offered the possibility to use the cosmetic product twice daily on affected skin over several months and were asked to return for control visits after 1 to 3 months. Data on the duration and previous therapies of MC were collected at the beginning of treatment, as were demographic data such as age and gender. The clinical status of MC infection was documented, and photos of representative lesions were taken at baseline and follow-up visits if consented by caregivers and children.

## 3. Results

**Figure 1:**
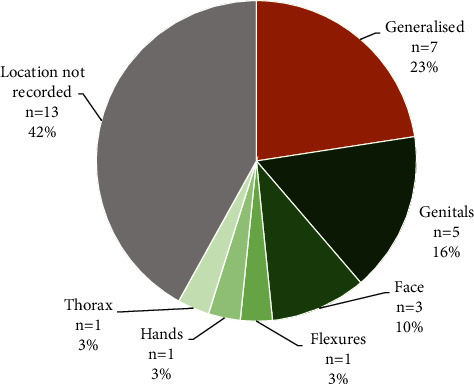
Localization of molluscum contagiosum (MC) lesions at baseline in *n* = 31 children presenting to a Portuguese Children's Hospital with MC as the primary complaint.

Portuguese children (*n* = 31; 15 male, 16 female; age 2 to 15 years (mean 6.2 years)) with active MC infection were included in this observational study. Typical MC lesions were localized to defined body areas or children presented with generalized disease (Figure 1). 6/31 (19.4%) children had previously received other topical therapies that were unsuccessful, and 25/31 (80.7%) were naïve to treatment.

**Figure 2:**
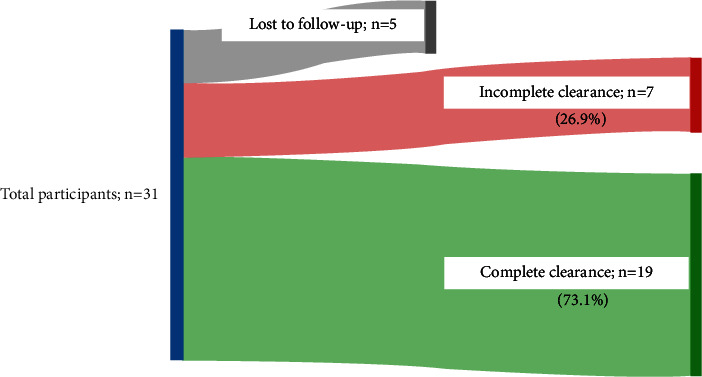
Outcome after an average treatment time with Spirularin^®^ VS cream twice daily of 4 months. Complete clearance refers to absence of any visible signs of active MC infections: patients with no complete clearance required additional or other treatment options. Percentages based on patients that were no lost to follow-up (*n* = 26).

**Figure 3:**
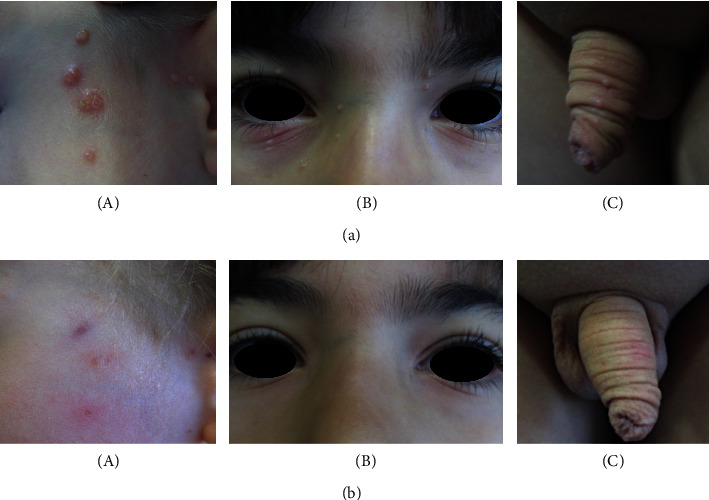
Examples for clinical responses in sensitive areas after twice daily application of Spirularin^®^ VS cream. (a) MC lesions before and (b) after treatment. (A), clearance of MC lesions on the left cheek of a 2-year-old boy after 9 weeks of therapy, (B), remission of MC lesions in a 7-year-old boy after 4 weeks of therapy, and (C), remission of genital MC lesions after 3 weeks of treatment in a 6-year-old boy.

Spirularin^®^ VS cream was applied twice daily over a period of 1 to 9 months (mean treatment duration 3.9 months). Five children (16% of *n* = 31) did not return for control visits. 19 of the 26 children (73.1%) in whom control visits were performed achieved complete clearance of MC lesions by application of Spirularin^®^ VS cream (Figure 2) after an average treatment time of 3.7 months. Clinical responses included remissions in sensitive areas such as the face and genital area (Figure 3). In 7/26 children (26.9%) with follow-up visits, complete remission was not observed with Spirularin^®^ VS cream alone and other therapies were used (Figure 2, *n* = 6 patients received 5% potassium hydroxide (Molutrex^®^); *n* = 1 patient was treated with curettage). There was no clinical evidence of bacterial skin infection during treatment, and all children tolerated the topical application of Spirularin^®^ VS cream very well with no irritative skin reactions or unpleasant symptoms being observed or reported.

## 4. Discussion

MC infections are frequent, occur mostly in children, manifest often in difficult to treat areas such as the genital area and face, can cause infections over several weeks with spread by autoinoculation, and may be complicated by bacterial superinfection and ultimately scar formation. Topical treatments that have been tried for MC include podophyllotoxin, imiquimod, sodium nitrite, myrtle leaf extract, phenol, combinations of salicylic and lactic acid, potassium hydroxide, and vesicant cantharidine [10], most of which are irritating. Laser therapy or curettage can be an option but is also difficult in sensitive areas, cases with more widespread lesions, and in small children. While two agents, a topical formulation of cantharidine in a proprietary applicator and the nitric oxide-releasing berdazimer [14], are currently undergoing review for potential approval by the US Food and Drug Administration, there remains a high need for safe, well-tolerated, effective therapies that also ideally prevent bacterial infection.

Spirularin^®^ VS cream contains a specific antimicrobial extract derived from *Spirulina platensis* termed Spiralin^®^ and antivirally active polysaccharide Ca-SP [13]. In a previous study, a cream containing Spiralin^®^ and Ca-SP has been shown to successfully prevent lip herpes and to reduce crust formation and lip dryness [13]. Spiralin^®^ has also demonstrated potent and broad antibacterial effects *in vitro* against bacteria relevant for skin infections including *Staphylococcus aureus* [15]. It has been available in Europe since 2010 and has been proven to be very well tolerated (Ocean Pharma, Reinbek, Germany; data on file).

The promising clinical effects noted in this observational study even in sensitive skin areas mostly after 2 to 8 weeks of treatment warrant replication in controlled studies. Mechanistical studies have shown that the antiviral activity of Ca-SP in the case of HSV-1 and Kaposi's sarcoma-associated herpesvirus/human herpes virus 8 is likely based on the inhibition of attachment, i.e., the initial binding of virus particles to the membrane of host cells [13]. Since mature virions and extracellular virions, the two infectious particles of MCV, also attach to host cells *via* binding to proteoglycans, we speculate that interference of Ca-SP and/or other polysaccharides contained in the microalga extract with the attachment of MCV to keratinocytes contribute to the observed clinical effect.

In light of the previous documentation of prophylactic effects against herpes labialis and the clinical effects and good tolerability recorded in this open-label observational study, we propose Spirularin^®^ VS cream as a novel cosmeceutical treatment option for children with active MC lesions and recommend further studies in this and other viral skin diseases.

## Data Availability

All data supporting the results of the study are in the manuscript and can be made available from the corresponding author (Professor Kristian Reich; k.reich@uke.de) upon request.

## References

[1] Olsen J. R., Gallacher J., Piguet V., Francis N. A. (2014). Epidemiology of molluscum contagiosum in children: a systematic review. *Family Practice*.

[2] Olsen J. R., Piguet V., Gallacher J., Francis N. A. (2016). Molluscum contagiosum and associations with atopic eczema in children: a retrospective longitudinal study in primary care. *British Journal of General Practice*.

[3] Silverberg N. B. (2018). Molluscum contagiosum virus infection can trigger atopic dermatitis disease onset or flare. *Cutis*.

[4] Manti S., Amorini M., Cuppari C. (2017). Filaggrin mutations and Molluscum contagiosum skin infection in patients with atopic dermatitis. *Annals of Allergy, Asthma, & Immunology*.

[5] Peterson A. R., Nash E., Anderson B. (2019). Infectious disease in contact sports. *Sport Health*.

[6] Koopman R. J., Merrienboer F., Vredden S., Dolmans W. M. (1992). Molluscum contagiosum; a marker for advanced HIV infection. *British Journal of Dermatology*.

[7] Leung A. K. C., Barankin B., Hon K. L. E. (2017). Molluscum contagiosum: an update. *Recent Patents on Inflammation & Allergy Drug Discovery*.

[8] Meza-Romero R., Navarrete-Dechent C., Downey C. (2019). <p>Molluscum contagiosum: an update and review of new perspectives in etiology, diagnosis, and treatment</p&gt. *Clinical Cosmetic and Investigational Dermatology*.

[9] Schaffer J. V., Berger E. M. (2016). Molluscum contagiosum. *JAMA Dermatol*.

[10] Phan S., Wyant C., Huynh C., Joaquin C., Hassan O. (2021). Efficacy of topical treatments for molluscum contagiosum in randomized controlled trials. *Clinics in Dermatology*.

[11] Gerlero P., Hernandez-Martin A. (2018). Update on the treatment of molluscum contagiosum in children. *Actas Dermo-Sifiliográficas*.

[12] van der Wouden J. C., van der Sande R., Kruithof E. J., Sollie A., van Suijlekom-Smit L. W., Koning S. (2017). Interventions for cutaneous molluscum contagiosum. *Cochrane Database of Systematic Reviews*.

[13] Mader J., Gallo A., Schommartz T. (2016). Calcium spirulan derived from Spirulina platensis inhibits herpes simplex virus 1 attachment to human keratinocytes and protects against herpes labialis. *Journal of Allergy and Clinical Immunology*.

[14] Browning J. C., Enloe C., Cartwright M. (2022). Efficacy and safety of topical nitric oxide-releasing berdazimer gel in patients with molluscum contagiosum: a phase 3 randomized clinical trial. *JAMA Dermatol*.

[15] Imhoff J. (2013). Wirkkosmetik für Haut und Nägel bei gestörter Hautbarriere. *Asthetische Dermatologie*.

